# Feasibility of combination chemotherapy with cisplatin and etoposide for haemodialysis patients with lung cancer

**DOI:** 10.1038/sj.bjc.6600687

**Published:** 2003-01-28

**Authors:** R Watanabe, Y Takiguchi, T Moriya, S Oda, K Kurosu, N Tanabe, K Tatsumi, K Nagao, T Kuriyama

**Affiliations:** 1 Department of Respirology (B2), Graduate School of Medicine, Chiba University, 1-8-1 Inohana, Chuo-ku, Chiba 260-8670, Japan; 2 Department of Emergency and Critical Care Medicine (J3), Graduate School of Medicine, Chiba University, Chiba, Japan; 3 Health Sciences Center, Chiba University, Chiba, Japan

**Keywords:** haemodialysis, chemotherapy, renal insufficiency, cisplatin, etoposide

## Abstract

Cancer chemotherapy for haemodialysis patients has never been established. To elucidate the feasibility of cisplatin-based combination chemotherapy for haemodialysis patients with lung cancer, a dose escalation study was conducted. Five haemodialysis patients with lung cancer were treated with cisplatin and etoposide. A starting dose of 40 mg m^−2^ of cisplatin on day 1 and 50 mg m^−2^ of etoposide on days 1, 3 and 5 were administered as the first course for the first patient. Membrane haemodialysis was regularly performed three times a week and soon after the completion of therapy. By monitoring toxicity and pharmacokinetics data, the dose was escalated course by course and patient by patient. Dose escalation was completed for the first two patients resulting in full-dose chemotherapy consisting of 80 mg m^−2^ of cisplatin on day 1 and 100 mg m^−2^ of etoposide on days 1, 3 and 5. Multiple courses of the full-dose chemotherapy were administered to the other three patients. Toxicity was manageable and tolerable for all. Pharmacokinetics data were comparable to those from patients with normal renal function, except for potential long-lasting higher levels of free platinum in the renal insufficiency group. In conclusion, this standard-dose combination chemotherapy was feasible even for haemodialysis patients.

Recent advances in haemodialysis for patients with renal insufficiency have resulted in longer survival than ever. Consequently, they have equal or increased risk of suffering from various neoplastic diseases, including primary lung cancer (Maisonneuve *et al*, 1999). A standard treatment for small-cell lung cancer and advanced nonsmall-cell lung cancer is the cisplatin-based combination chemotherapy (Evans *et al*, 1985; Johnson, 2000). However, as its principal elimination route is renal (Belt *et al*, 1979; Gormley *et al*, 1979), it has never been routinely administered to patients with renal insufficiency undergoing haemodialysis. Most previous studies on chemotherapy for such patients have reported the feasibility of cisplatin administration at lower than standard doses, in limited patient numbers (Ayabe *et al*, 1989; Umeki *et al*, 1990; Ono *et al*, 1992). Chemotherapy consisting of such low doses of cisplatin, however, has never been shown to be sufficiently effective for lung cancer, either the small-cell or nonsmall-cell variety.

To determine adequate dose levels of the combination chemotherapy of cisplatin and etoposide for haemodialysis patients, a dose escalation study was conducted in a limited number of patients under the guidance of pharmacokinetics monitoring.

## Patients and Methods

### Patients

Five patients with lung cancer and renal insufficiency undergoing haemodialysis, three with stage IV or recurrent adenocarcinoma and two with small-cell lung carcinoma, were enrolled in this study. Characteristics of the five patients are listed in Table 1

**Table 1:** Patient characteristics

**Case no.**	**Gender**	**Age**	**Histology^a^**	**Clinical stage**	**PS^b^**	**Cause of renal failure^c^**	**Duration of haemodialysis (year)**
Case 1	Male	74	Sm	T2 N1 M0	1	MPGN	2
Case 2	Female	43	Ad	T3 N2 M1	0	IgA	12
Case 3	Male	58	Sm	T4 N3 M1	2	DM	6
Case 4	Male	72	Ad	recurrence	0	Unknown	1
Case 5	Male	50	Ad	T4 N2 M1	0	DM	5

a Sm, small-cell lung cancer; Ad, adenocarcinoma of lung.

b PS, performance status (ECOG).

c MPGN, membranoproliferative glomerulonephritis; IgA, IgA nephropathy; DM, diabetic nephropathy.

. Three other patients with small-cell lung cancer and normal renal function were enrolled for pharmacokinetics comparison. Written informed consent was obtained from all the patients. The study fully complied with institutional regulations. Two of the five patients had previous cancer therapies. Case 4 ( Table 1) had resection and radiotherapy of laryngeal carcinoma 8 years earlier, resection of primary lung cancer 5 years earlier, and radiotherapy for pulmonary recurrence 1 year before the second pulmonary recurrence of lung cancer and enrolment in the study. Case 5 ( Table 1) had trans-urethral resection of bladder cancer 2 years before the development of primary lung cancer and enrolment in the study. The other three patients had had no prior chemotherapy or radiotherapy.

### Haemodialysis

All five patients with renal insufficiency had been maintained with regular three-times-a-week haemodialysis utilising membrane filters before entering the study. From the start of the study, membrane haemodialysis was continued with the exclusive use of high-performance membranes (BS series, TORAY Medical Co., Tokyo) three times a week, with a single haemodialysis lasting approximately 4 h. On the days of chemotherapy, haemodialysis was started within 10 min after completion of the administration of the agents.

### Chemotherapy and dose escalation

The chemotherapeutic regimen for the eight patients, the five haemodialysis patients and the three patients with normal renal function, consisted of cisplatin on day 1 and etoposide on days 1, 3 and 5, every 4 weeks. The schedule of the administration of the two agents on day 1 consisted of intravenous injection of etoposide in 500 ml of normal saline over 60 min, and of cisplatin over 30 min starting 30 min after the start of etoposide infusion, resulting in the simultaneous completion of the two agents. Hydration with 2000 ml of electrolyte solution, on the day before day 1 and with 3000 ml of electrolyte solution on day 1, was performed for the patients with normal renal function. No hydration was done for the five haemodialysis patients. All patients received granisetron hydrochloride, 6 mg i.v. in two fractions, for nausea and vomiting prophylaxis on day 1. For the three patients with normal renal function, standard-dose chemotherapy was administered, consisting of cisplatin and etoposide at 80 and 100 mg m^−2^, respectively. For the haemodialysis patients, half-dose chemotherapy (40 mg m^−2^ of cisplatin on day 1 and 50 mg m^−2^ of etoposide on days 1, 3 and 5) was administered for the first course of the first patient of this group (Case 1, Table 1), based on previously published reports that these doses were safe even for such patients (Ono *et al*, 1992; Yanagawa *et al*, 1996). Thereafter, the dose escalation protocol consisted of: (1) pharmacokinetics analysis of every course immediately after its completion, (2) course-by-course dose escalation to determine maximum tolerable dose (MTD), but not to exceed the standard doses for the patients with normal renal function, (3) initial dose escalation of cisplatin alone with reference to pharmacokinetics comparison between the previous courses and average data from the three patients with normal renal function, (4) dose escalation of etoposide, again with reference to the pharmacokinetics comparison between the two, (5) one more repetition of the same dose when dose limiting toxicity (DLT) is observed, and (6) determination of MTD when DLT is observed in two successive courses. DLT is defined as any of grade 4 haematological toxicity except anaemia and grade 3 or higher nonhaematological toxicity except nausea/vomiting and alopecia. Toxicity caused by the treatment was evaluated according to the National Cancer Institute Common Toxicity Criteria version 2.0.

### Pharmacokinetics study

Venous blood samples were collected into heparinised tubes immediately upon the completion of drug administration, then at 0.5, 1, 2, 3, 4, 5, 8, 16 and 24 h on days 1, 3 and 5, followed by additional once-a-day sampling on days 7, 14 and 28. After separating plasma from each sample, concentrations of total platinum (t-Pt) and etoposide were measured in a portion of the plasma. The rest was ultrafiltered by centrifuging at 3000 rpm for 20 min with a filter (Amicon Centrifree MPS-3, Millipore Corp., Bedford, MA, USA) and was used for measurement of nonprotein-bound platinum or free platinum (f-Pt). Separated plasma was stored at −80°C until measurement. Concentrations of t-Pt and f-Pt were determined by flameless atomic absorption spectrophotometry (LeRoy *et al*, 1977), and those of etoposide were determined by high-performance liquid chromatography (Strife *et al*, 1981). Pharmacokinetics data were analysed according to the previously reported method (Yamaoka *et al*, 1978). Briefly, all measurements before the next administration of the same agent were used for calculations of pharmacokinetics parameters, unless each measurement was less than 5% of the maximum concentration (*C*_max_). When a measurement before the next administration was less than 5% of *C*_max_, only measurements before this time point were used for the calculation of the data.

### Dialysability of cisplatin and etoposide

At 10 min after the start of each haemodialysis, blood samples were collected from both import and export sides of the dialyser for the measurements of f-Pt, t-Pt and etoposide concentrations. Then, the *in situ* elimination rate of each agent with the dialysers was determined by the following formula: elimination rate=(concentration in the import side − concentration in the export side)/concentration in the import side.

### Evaluation of tumour response

Objective tumour response was evaluated according to the ‘Response evaluation criteria in solid tumors’ guideline (Therasse *et al*, 2000), and the response was classified into the four categories: complete response, partial response, stable disease and progressive disease.

### Statistical analysis

Comparisons of each pharmacokinetics parameter between patient groups and dose groups were analysed by Student's unpaired *t*-test. Comparisons of each pharmacokinetics parameter according to chemotherapeutic course number were performed with the Kruskal–Wallis test. Differences were judged as significant when *P* values were less than 0.05 (two-sided).

## Results

### Dose escalation of cisplatin and etoposide in haemodialysis patients

Dose escalation was completed in the first two cases, and the final dose level was the same as the standard doses for patients with normal renal function, that is, 80 mg m^−2^ of cisplatin and 100 mg m^−2^ of etoposide. The other three patients were treated with multiple courses of this dose level to confirm its feasibility. That is, based on tolerable toxicity and the results of pharmacokinetics (data shown below) with the initial dose (40 mg m^−2^ of cisplatin and 50 mg m^−2^ of etoposide) in the first course of the first patient, the dose of cisplatin was escalated to 80 mg m^−2^ with the etoposide dose being unchanged for the second course of the same patient (Case 1, Table 1). Then, the dose of etoposide was escalated to 100 mg m^−2^, with cisplatin being 40 mg m^−2^ in the first course of the second patient (Case 2). Again, based on tolerable toxicity and pharmacokinetics (data shown below), the standard doses of 80 mg m^−2^ of cisplatin and 100 mg m^−2^ of etoposide were administered in the second course of the same patient (Case 2). For the other three cases with renal insufficiency, this standard-dose chemotherapy was performed for four courses in Cases 3 and 5, and for two courses in Case 4, based on their medical requirements.

### Effects of chemotherapy

As for objective tumour response, partial response was obtained in four of the five cases (Cases 1, 3, 4 and 5). As Case 1 had a limited disease of small-cell lung cancer, curative-intent thoracic radiotherapy was performed after the completion of the two courses of chemotherapy.

### Toxicity

Toxicity is summarised in Table 2

**Table 2:** Toxicity^a^

**Patient**	**Course no.**	**Anaemia**	**Neutropenia**	**Thrombocytopenia**	**Nausea and vomiting**
Case 1	1	2	2	2	2
	2	3	3	3	2
					
Case 2	1	3	2	0	0
	2	3	3	0	2
					
Case 3	1	4	3	0	2
	2	4	2	0	2
	3	4	2	0	2
	4	4	2	0	2
					
Case 4	1	3	3	2	3
	2	4	3	3	3
					
Case 5	1	3	2	0	3
	2	3	3	0	3
	3	3	3	2	3
	4	3	2	2	3

a Graded by NCI-CTC Version 2.0.

. Briefly, all five haemodialysis patients experienced anaemia and neutropenia, and four required transfusion because of grades 3 and 4 anaemia. Grades 2 and 3 thrombocytopenia were observed in one and two of the patients, respectively. In Case 2, the start of the second course was postponed for 1 week because of lingering neutropenia and anaemia. In Case 3, the start of the third course was put off for 1 week because of lingering neutropenia.

As for nonhaematological toxicity, grade 3 nausea and vomiting was observed in Cases 4 and 5. On day 5 of the fourth course in Case 5, the administration of etoposide was skipped because of prolonged nausea. Other toxicity including liver dysfunction and pulmonary damage was not observed. Recovery from toxicity was complete in all the cases.

### Pharmacokinetics

**Figure 1:**
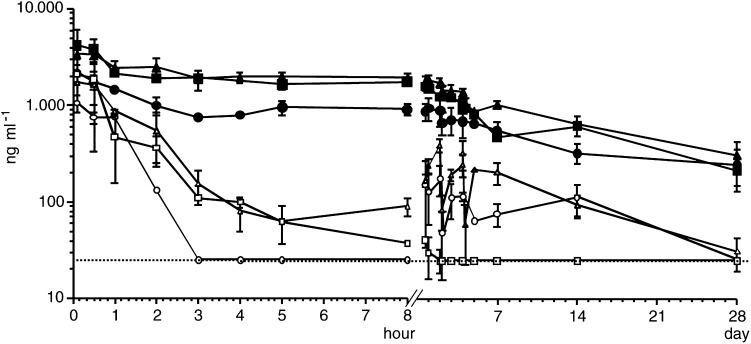
Time–concentration curves of platinum. After 30-min i.v. infusion of cisplatin at doses of 40 mg m^−2^ (*n*=2), 80 mg m^−2^ (*n*=5) for haemodialysis patients, or 80 mg m^−2^ (*n*=3) for patients with normal renal function, plasma concentrations of f-Pt and t-Pt were sequentially determined. Each dot and bar represent mean and standard deviation, respectively. Open and closed circles represent f-Pt and t-Pt, respectively, when 40 mg m^−2^ of cisplatin was administered to haemodialysis patients. Open and closed triangles represent f-Pt and t-Pt, respectively, when 80 mg m^−2^ of cisplatin was administered to haemodialysis patients. Open and closed squares represent f-Pt and t-Pt, respectively, when cisplatin was administered at 80 mg m^−2^ to patients with normal renal function. The lowest detection limit of f-Pt was 25 ng ml^−1^, and measurements below this value were plotted on the dotted line in this figure. Note that f-Pt levels were still detectable from days 2 to 14 in the haemodialysis patients, whereas those in patients with normal renal function were all below the detection limit at the same time points.

Although pharmacokinetics analysis was performed for every course of each patient and all data were monitored, only a data set from the first administration of a given dose level of an agent in each patient was analysed in this presentation to avoid a bias because of patient variation rather than dose variation. This bias would be inevitable because some patients were treated with more courses than others. Therefore, for creating the time–concentration curves in Figures 1

and 2

**Figure 2:**
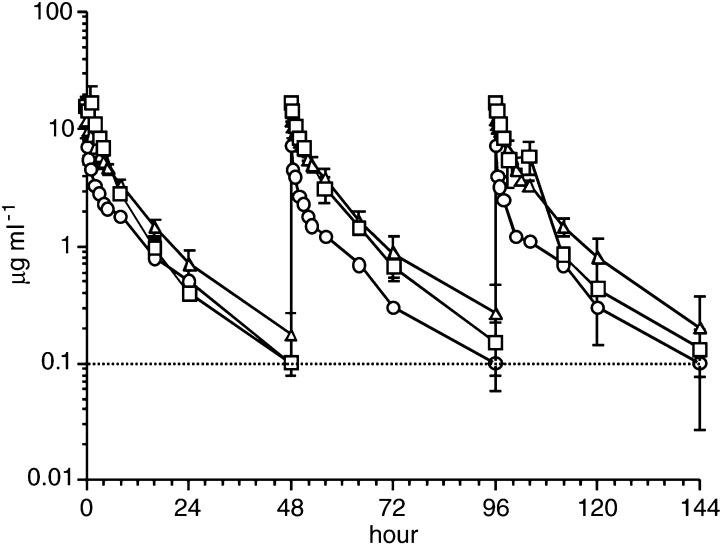
Time–concentration curves of etoposide. After 60-min i.v. infusion of etoposide at doses of 50 mg m^−2^ (*n*=1), 100 mg m^−2^ (*n*=4) for haemodialysis patients, or 100 mg m^−2^ (*n*=3) for patients with normal renal function, plasma concentrations of etoposide were sequentially determined. Each dot and bar represent mean and standard deviation, respectively. The circles, triangles and squares represent etoposide concentrations when etoposide was administered at 50 or 100 mg m^−2^ to haemodialysis patients, or at 100 mg m^−2^ to patients with normal renal function, respectively. The lowest detection limit of etoposide was 0.1 *μ*g ml^−1^, and measurements below this value were plotted on the dotted line in this figure.

, data from the first course of Cases 1 and 2 were used for the 40-mg m^−2^ cisplatin curves; data from the second course of Cases 1 and 2, and from the first course of Cases 3–5 were used for the 80-mg m^−2^ cisplatin curves; data from the first course of Case 1 were used for the 50-mg m^−2^ etoposide curve; data from the first course of Cases 2–5 were used for the 100-mg m^−2^ etoposide curves. The same data set was used for calculating the pharmacokinetics parameters according to the dose of the agent. For etoposide, mean values of each parameter of the three administrations in each course were used to represent the course. All data on cisplatin and all the first administrations of etoposide in each course, however, were used for the calculation of the pharmacokinetics parameters according to the course number. Thus, the pharmacokinetics parameters consisting of *C*_max_, half-time (*t*_1/2_), area under the curve (AUC), total clearance (Cl_tot_) and volume of distribution (*V*_dss_) of cisplatin and etoposide are summarised in Table 3

**Table 3:** Pharmacokinetics parameters of platinum

	**f-Pt**					**t-Pt**				
	***C*_max_ (*μ*g ml^−1^)**	***t*_1/2_ (h)**	**AUC (*μ*g ml^−1^ h)**	**Cl_tot_ (l h^−1^)**	***V*_dss_ (l)**	***C*_max_ (*μ*g ml^−1^)**	***t*_1/2_ (h)**	**AUC (*μ*g ml^−1^ h)**	**Cl_tot_ (l h^−1^)**	***V*_dss_ (l)**
Dose^a^ (mg m^−2^)										
40^b^ (*n*=2)	1.1±0.21	0.62±0.33	1.4±0.05	44.6±3.2	43.5±19.0	2.2±0.44	275.4±11.8	337±82	0.19±0.026	73.3±6.7
80^b^ (*n*=5)	1.9±0.55	0.82±0.18	2.7±0.82	48.5±15.4	58.4±17.9	3.5±0.51	267.2±60.8	652±98	0.19±0.044	68.1±16.0
80^c^ (*n*=3)	2.1±1.03	0.70±0.22	2.8±1.59	55.2±38.5	46.3±23.3	4.2±1.94	293.8±80.4	558±107	0.21±0.049	76.9±9.4
										
Course number^d^										
1st (*n*=3)	2.2±0.38	0.72±0.18	3.3±0.53	38.1±7.6	48.5±12.1	3.7±0.64	298.4±49.7	686±63	0.18±0.017	71.6±21.0
2nd (*n*=3)	2.4±0.07	0.66±0.09	3.2±1.07	41.5±13.8	43.7±11.5	4.1±0. 71	329.1±133.2	873±99	0.14±0.015	62.4±19.7
3rd (*n*=2)	2.4±0.00	0.79±0.06	3.4±0.79	37.5±10.2	44.6±11.1	3.8±0.68	368.4±25.9	893±44	0.14±0.012	72.2±0.4
4th (*n*=2)	1.9±1.19	0.92±0.32	2.8±0.79	45.3±11.1	69.1±39.0	3.8±1.24	528.2±180.2	1268±294	0.10±0.019	71.7±8.4

a Pharmacokinetics parameters according to dose and patient population.

b For haemodialysis patients.

c For patients with normal renal function.

d Pharmacokinetics parameters according to course number, in which 80 mg m^−2^ cisplatin was administered, in haemodialysis patients. Mean±s.d.

and 4

**Table 4:** Pharmacokinetics parameters of etoposide

	***C*_max_ (*μ*g ml^−1^)**	***t*_1/2_ (h)**	**AUC (*μ*g ml^−1^ h)**	**Cl_tot_ (l h^−1^)**	***V*_dss_ (l)**
Dose^a^ (mg m^−2^)					
50^b^ (*n*=1)	7.1	11.4	45.1	1.9	25.3
100^b^ (*n*=4)	12.3±1.63	10.8±3.78	91.7±11.7	1.7±0.25	19.2±5.07
100^c^ (*n*=3)	16.1±2.66	6.3±0.33	93.7±9.3	1.5±0.09	10.4±0.73
					
Course number^d^					
1st (*n*=4)	12.3±1.63	10.8±3.78	91.7±11.7	1.7±0.25	19.2±5.07
2nd (*n*=4)	11.2±1.83	12.9±3.69	95.7±31.9	1.8±0.69	22.9±5.46
3rd (*n*=2)	10.5±1.41	13.5±4.31	106.7±65.2	1.8±1.12	22.0±3.37
4th (*n*=2)	10.2±1.76	24.1±18.7	125.3±69.9	1.5±0.81	29.2±9.65

a Pharmacokinetics parameters according to dose and patient population.

b For haemodialysis patients.

c For patients with normal renal function.

d Pharmacokinetics parameters according to course number, in which 100 mg m^−2^ etoposide was administered, in haemodialysis patients. Mean±s.d.

, respectively. In these tables, the parameters obtained from the five patients with renal insufficiency were compared to those from the three patients with normal renal function. Figures 1 and 2 also compare the concentration–time curves of cisplatin and etoposide between the patients with renal insufficiency and with normal renal function.

*C*_max_ and AUC of f-Pt, t-Pt and etoposide were similar between the two patient groups when the same doses of the agents were administered, and showed potentially lower levels (approximately half) than those of half-dose administration to the haemodialysis patients, although only differences in *C*_max_ and AUC of t-Pt had statistical significance (*P*=0.024 and 0.011, respectively). *t*_1/2_, Cl_tot_ and *V*_dss_ of f-Pt and t-Pt were also similar between the two patient groups. *V*_dss_ of etoposide in the haemodialysis patients was significantly higher than in the patients with normal renal function when the same dose (100 mg m^−2^) was administered (*P*=0.034).

Tables 3 and 4 also compare the pharmacokinetics data according to the course number of chemotherapy in the haemodialysis patients. Repeated administration showed a tendency to cause increased AUC and decreased Cl_tot_ in t-Pt (*P*=0.054 and 0.052, respectively), but not in f-Pt (*P*=0.93 and 0.85, respectively) or etoposide (*P*=0.97 and 0.97, respectively).

### Dialysability of cisplatin and etoposide

Calculated dialysabilities of f-Pt and t-Pt were 86.5±22.1 (*n*=13) and 44.0±12.0% (*n*=14), respectively. In contrast, the calculated dialysability of etoposide was relatively low at 13.0±12.6% (*n*=39).

## Discussion

As many of the chemotherapeutic agents for cancer involve renal excretion, cancer chemotherapy for patients with renal insufficiency undergoing haemodialysis has never been well established. Several pilot studies concerning the use of certain chemotherapeutic agents including cisplatin for haemodialysis patients, however, have shown the feasibility of such attempts by reducing the doses of the agents (Ayabe *et al*, 1989; Umeki *et al*, 1990; Ono *et al*, 1992). Contiguity of therapeutic and toxic dose ranges of the chemotherapeutic agents, however, casts doubt on the effectiveness of such significantly dose-reduced chemotherapy. Contrary to those previous studies, our study demonstrated the feasibility of full-dose combination chemotherapy with cisplatin and etoposide in lung cancer patients undergoing haemodialysis.

In this study, half doses of cisplatin and etoposide of the standard amount were administered in the first course of Case 1, because these doses were reportedly safe even for such patients (Ono *et al*, 1992; Yanagawa *et al*, 1996). Toxicity in the course was well tolerated. Following this, comparison between the pharmacokinetics data of this course, the pharmacokinetics data obtained from the three patients with normal renal function, and previously published data (Wakui *et al*, 1986; Kitajima *et al*, 1987) suggested that these agents could be doubled in dose. Despite this, however, only cisplatin was escalated to the standard dose in the second course of the same patient and, thereafter, only etoposide was escalated to the standard dose, cisplatin being half of the standard, in the first course of the second patient. Monitoring of the toxicity and pharmaco-kinetics data during these chemotherapeutic courses again suggested the feasibility of the standard-dose combination of the agents. As a result, multiple administrations of full-dose combination chemotherapy, ranging from two to four courses, were given to the other three patients. In these patients, toxicity was tolerable and pharmacokinetics data were comparable to the data obtained from the three patients with normal renal function, suggesting the safety of this full-dose combination chemotherapy for haemodialysis patients. A typical dose escalation test for cancer chemotherapy usually employs a certain dose level for three to five patients and when DLT is observed in less than one-third or half of the patients, the next dose level is administered to the next set of patients. Contrary to this typical method, the present study performed course-by-course dose escalation under the guidance of pharmaco-kinetics analysis. Since the standard dose of this regimen has been well established for patients with normal renal function, comparison between pharmacokinetics data obtained from the patients with normal renal function and course-by-course pharmacokinetics data from the haemodialysis patients enabled us to accomplish this dose escalation test in such a small patient number.

Tumour control by this regimen was also satisfactory, with two partial responses in two patients with small-cell lung cancer and two partial responses in three patients with nonsmall-cell lung cancer. Similar dose escalation studies were also reported previously (Holthuis *et al*, 1985; Fox *et al*, 1991), but they involved a single agent for dose escalation, in contrast to ours, which used a combination of two agents and their escalated doses. Holthuis *et al* (1985) performed combination chemotherapy with cyclophosph-amide, adriamycin and etoposide for haemodialysis patients with small-cell lung cancer, and a gradual dose escalation of etoposide showed that 127 mg m^−2^ of the agent was feasible. Fox *et al* (1991) conducted a dose escalation study with cisplatin, combined with 500 mg m^−2^ of cyclophosphamide, for haemodialysis patients with seminoma and showed that 100 mg m^−2^ of cisplatin was feasible.

Although mechanisms underlying these results have not been completely understood, the efficient dialysability of f-Pt shown in the present study may partly explain this phenomenon. As f-Pt is efficiently cleared by haemodialysis, protein-bound platinum might be efficiently decomposed to f-Pt in the body. Thus, both f-Pt and t-Pt might have been cleared as well as in the patients with normal renal function. Quite contrary to cisplatin, the dialysability of etoposide was low in this study, while clearance of the agent in the haemodialysis patients was as efficient as that in patients with normal renal function. Although this observation is similar to previously published data (Holthuis *et al*, 1985; Sauer *et al*, 1990; Stewart, 1994), it seems at variance with the excretion routes of this drug in patients with normal renal function, where it is reportedly normally eliminated by renal (60%) and hepatic (40%) mechanisms (English *et al*, 1996). Our observation could be explained by the assumption that the hepatobiliary route completely compensates for the renal route in haemodialysis patients, but our study did not present any evidence to support this hypothesis. The increased *V*_dss_ of etoposide in the haemodialysis patients suggested by this study might be a consequence of such altered excretion route. In any case, the data on dialysability of f-Pt, t-Pt and etoposide in this study were concordant with those of a previous study (Sauer *et al*, 1990).

Although all toxicity was tolerable, frequent anaemia and thrombocytopenia prolonged in some cases were observed. As a result of the tendency of a gradual increase in AUC and a decrease in Cl_tot_ of t-Pt after multiple administration, toxicity would become a more critical issue if the chemotherapy were repeated within a short period.

In conclusion, a dose escalation study was conducted in a small patient population together with pharmacokinetics monitoring, and multiple administration of the full-dose combination chemo-therapy comprising cisplatin and etoposide was shown to be possible in haemodialysis patients. Since this study was based on only five patients, further studies employing the same regimen for larger patient populations are indeed warranted. In addition, different regimens containing other recently developed agents could certainly be investigated in a similar manner. Further studies on optimal chemotherapy for haemodialysis patients with malignant diseases are also called for.
